# Bayesian dynamic network actor models with application to South Korean COVID-19 patient movement data

**DOI:** 10.1007/s10260-021-00599-x

**Published:** 2021-10-22

**Authors:** Antonio Mario Arrizza, Alberto Caimo

**Affiliations:** 1 University of Bologna, Bologna, Italy; 2 Technological University Dublin, Dublin, Ireland

**Keywords:** COVID-19 patient movements, Relational events, Dynamic network actor models, Bayesian inference

## Abstract

Motivated by the ongoing COVID-19 pandemic, this article introduces Bayesian dynamic network actor models for the analysis of infected individuals’ movements in South Korea during the first three months of 2020. The relational event data modelling framework makes use of network statistics capturing the structure of movement events from and to several country’s municipalities. The fully probabilistic Bayesian approach allows to quantify the uncertainty associated to the relational tendencies explaining where and when movement events are established and where they are directed. The observed patient movements’ patterns at an early stage of the pandemic can provide interesting insights about the spread of the disease in the Asian country.

## Introduction

The first case of COVID-19 infection in South Korea was reported on January 20, 2020 and was followed by a steady increase in the number of cases over the following month which made South Korea one of the first hardest-hit countries by the virus. By the end of March 2020, South Korea was successfully able to control the epidemic wave which reached its peak on March 11, 2020.

This paper is motivated by the recent publication of COVID-19 infected patient movement dataset collected from January 20, 2020, to May 31, 2020 by the Korean Center for Disease Control and Prevention (KCDC). The COVID-19 patient movement events are directly observable and do not need to be aggregated into valued network structures. This facilitates the use of statistical models that can reflect their data-generating process. Advanced statistical network analysis methods provide with the possibility of characterising mobility patterns by evaluating different scenarios in the evolution of the pandemic and potential countermeasures to mitigate the impact of future infection waves.

The dynamic network actor model (DyNAM) framework Stadtfeld et al (2017) for relational event data allows to investigate the main network effects that describe the origin, time and direction of the movement events by differentiating between two sub-models modelling the waiting times until an event is initiated and the choice of its destination. The network embeddedness and the role played by large Korean municipalities in this dataset are examples of how frequent connections may explain the spread of the disease across the entire country.

A dynamic network can be represented via a time-indexed graph $Y(t) = (N(t), X(t))$ with *t* varying, where *N*(*t*) is the node set present in the network at time *t* and *X*(*t*) represents the set of values indicating the presence (and potentially the value) of the edges connecting nodes at a certain time point. More formally, we defined the binary dyadic variable:

$$x_{{ij}} (t) = \left\{ {\begin{array}{*{20}l} {1,} \hfill & {{\text{if}}\;{\text{there}}\;{\text{is}}\;{\text{an}}\;{\text{edge}}\;{\text{between}}\;{\text{node}}\;i\;{\text{and}}\;{\text{node}}\;j\;{\text{at}}\;{\text{time}}\;t;} \hfill \\ {0,} \hfill & {{\text{otherwise}}.} \hfill \\ \end{array} } \right.$$

Dynamic networks change over time either in terms of covariates (nodal or dyadic) or tie formation. In the first case, we are interested in modelling a dynamic changes *of* the network. In the second case we are interested in modelling the dynamic changes *on* a network (Kolaczyk 2017).

Dynamic networks can be either in the form of panel data i.e., consisting of network snapshots observed at discrete time points or instantaneous data i.e., consisting of fine-grained network changes. The two core statistical models that study longitudinal network data are temporal exponential random graph models (TERGMs) (Hanneke et al 2010) and stochastic actor-oriented models (SAOMs) (Snijders et al 2010). TERGMs are natural mathematical extension of the family of exponential random graph models (ERGMs) (Holland and Leinhardt 1981; Frank and Strauss 1986). By jointly modelling network states at multiple time points, TERGMs consider the probability of observing a network at time *t* as a function of networks formed up to a previous time and to the network statistics usually accounted for in ERGMs (for a list of such statistics see Morris et al (2008)). SAOMs are special cases of the continuous-time Markov modeling framework of Holland and Leinhardt (1977) modelling tie changes between network states at consecutive time points by assuming that these states are repeated snapshots of a Markov process evolving in continuous time defined by transition probabilities determined by an unobserved sequence of mini-steps involving tie changes. Longitudinal ERGMs (Koskinen and Snijders 2007; Koskinen et al 2015) represent another important family of network models for dynamic network data that assume the existence of a continuous-time Markov ERGM process describing the evolution of relational data.

Panel data are often easier to collect. Observing a full graph at discrete points in time is not rare, whereas, collecting the information about the exact time point a node gets active is not easy. However, the latter type of data sources brought the attention to model for relational events or counting processes (Hunter 2019) which are extensions of survival models applied to network data. More precisely, a relational event is a discrete directed tie generated by a node (sender) and directed towards another node (receiver), the sender set can either equal the receiver set or not (Butts 2008). By building on event history analysis, the goal of such models is to combine relational dependencies and temporal dynamics to find meaningful network mechanisms (Brandenberger 2020).

The most important models apt to analyse relational event data are relational event models (REMs) (Butts 2008) and dynamic network actor models (DyNAMs) Stadtfeld et al (2017), whose difference lies in their ‘orientation’. REMs formulate the probability of observing a tie at a specific time given a set of tie-oriented statistics. DyNAMs assume conditional independence between two separable processes given the observed data: the activity rate sub-model of the sender of the event and the choice sub-model of the receiver of the event given a set of actor-oriented network statistics. The aim of the researcher while using them is, but not limited to, explain the temporal order of ties within a connected environment. Why do two countries start to sign a treaty (Stadtfeld and Block 2017)? What drives the inter-hospital patient movements within a certain region (Vu et al 2017)? Why do conflict or cooperation events occur over water consumption (Hollway 2020)?

In this paper, we focus on the implementation of Bayesian DyNAMs for the analysis of South Korea COVID-19 patient movement relational event data. Although it has not been explored yet, a Bayesian treatment of this family of models is appropriate as it allows to quantify the uncertainty of the parameters associated to the network effects by building a fully probabilistic inferential framework. Furthermore, expert knowledge evaluation and validation can be incorporated by specifying adequate priors before collecting the data.

The paper is organized as follows. In Sect. 2, we give a description of the South Korea COVID-19 patient movement dataset. In Sect. 3, we describe the main statistical properties of DyNAMs. In Sect. 4 we give a brief overview of the network statistics and covariates that will help us describe the dynamics of relational event data. In Sect. 5, we present the Bayesian simulation procedure for estimating DyNAM posterior parameter distribution via a Metropolis-Hastings algorithm (Hastings 1970; Metropolis et al 1953). In Sect. 6, we demonstrate the Monte Carlo inferential procedure by analysing the well-known Social Evolution dataset. This example is meant to detail the use of prior specification in a context where previous information about the main network effects characterising the event dynamics is available from previous literature. In Sect. 7, we carry out the COVID-19 patient movement data analysis. Lastly, in the final section, we draw some conclusions.

## South Korea COVID-19 patient movement data

South Korea was one of the first countries to experience a COVID-19 disease outbreak, caused by Severe Acute Respiratory Syndrome Coronavirus 2 (SARS-CoV-2). Its first case, imported from Wuhan, China, was reported on January 20, 2020. The daily number of confirmed cases varied from 0 to 2 per day for the first month of the outbreak, until a cluster was identified in the Daegu metropolitan area. The cluster originated from a patient who traveled around the cities of Daegu and Seoul before her diagnosis. Since then, daily infected patient numbers grew rapidly reaching a peak on February 29 at 909. Thereafter, the number of daily infections stabilised below 200, with 100 individuals on 25 March 2020.^1^

South Korea did not put into force strong lockdown strategies as other developed countries did. They operated by setting up a system of screening and diagnosis – two weeks from the first confirmed case, they shipped up to 100, 000 diagnosis kits per day^2^ – and of contact tracing – special hired officers were allowed to track and isolate infected individuals and their contacts by means of GPS data, credit card information and medical facility visits.^3^

Our analysis of this dataset will be designed to build a fully probabilistic relational event modelling approach capable of describing the propensity of COVID-19 patient movement event sequences among Korean municipalities based on some geographic covariate data.

## Dynamic network actor models for relational events

Dynamic actor network models (DyNAMs) are probabilistic models for relational event data - i.e., vectors formed by tuples of the form $a = (i,j,t)$ where $i, j {\text { and }} t$ represent, respectively, the event sender, receiver and time of the event. The collection of such tuples constitutes the time-ordered sequence $A_t$ up to time *t*.

The goal of DyNAMs is to explain the tendency of nodes to create events which may depend on global or local covariates, past events or current network configurations at given times. Stadtfeld et al (2017) define a time dependant process state as:

1 $$\begin{aligned} y(t) = (x^{(1)}(t),x^{(2)}(t),...,z^{(1)}(t),z^{(2)}(t),...,N^{(1)}(t),N^{(2)}(t),...) \end{aligned}$$

where *x*(*t*) denotes the network (endogenous) information, *z*(*t*) denotes the (exogenous) information represented by the covariates at a global or nodal level and *N* is the set of the nodes of the network.

DyNAMs are actor-oriented in nature: changes in the network ties creation are modelled as node characteristics (e.g., the presence of a covariate, the past activity of the node).

DyNAMs consist of two steps. In the first step, the waiting time until a sender gets involved is modeled. In the second step, the selection of a receiver is modelled conditionally on the selection of the sender. A significant assumption is the conditionally independence between the two steps given all the relevant information. More formally, DyNAMs treat the waiting time between one event to the next to be conditionally exponentially distributed via a composite Poisson rate

2 $$\begin{aligned} \phi _{ij}(y;\beta ,\theta ) = \mu _i(y;\beta ) \ \times \ p(i \rightarrow j,y \mid \theta ). \end{aligned}$$

The first term on the right side is the exponential hazard of the *i*-th node to become active – the waiting time of the sender to get active depends on a set of parameters $\beta :$

3 $$\begin{aligned} \mu _i(y;\beta ) = \exp \left\{ \beta ^{\top } r(i,y)\right\} , \end{aligned}$$

where the first element of the parameter vector $\beta$ is the intercept $\beta _0$ which measures the baseline tendency of the *i*-th node to send a connection, and $r(\cdot )$ is the statistic vector capturing the relational structure of the nodes in the network. In the first step of the modelling process, the node *i* with the highest rate $\mu _i(y;\beta )$ at a certain time *t* is the node with the highest probability of getting active at that time point and it is therefore selected as a sender of the directed event.

Based on this assumption, we can then construct the likelihood of timings and senders of a specific event sequence $A_t$, considering the cumulative number of events to be *M*. The likelihood is the product of the individual event likelihoods which are defined as the product of the sender’s hazard function and the survival functions of all the other senders over the time period between two consecutive events:

4 $$\begin{aligned} L (\beta )=\prod _{m=1}^M\mu _{i_m}(y_m ;\beta ) \prod _{i\in N_m}\exp \left\{ -\mu _i(y_m;\beta )(t_m-t_{m-1})\right\} . \end{aligned}$$

The second step of the modelling process focus on identifying the receiver *j* of the action created by the sender *i*. The probability of *j* being a receiver of the event is based on a multinomial probability distribution depending on all the relevant information – the exogenous/endogenous process state defined in Equation 1 – and on a set of parameters $\theta$:

5 $$\begin{aligned} p(i \rightarrow j,y \mid \theta ) = \frac{\exp \left\{ \theta ^\top s(i,j,y)\right\} }{\sum _{j \in N\setminus \{i\}}\exp \left\{ \theta ^\top s(i,j,y)\right\} }, \end{aligned}$$

where *s*(*i*, *j*, *y*) are the network statistics that characterise the connection $i \rightarrow j$ whose knowledge is inherited from the actor-level structure of the statistics used to capture the network dependence within the SAOM framework. In fact, dependence between ties is modeled by allowing the actors’ choices concerning tie changes to be influenced by local relational configurations. These statistics are similar to the ERGM ones but take the perspective of an actor-oriented formulation (Block et al 2019).

Again, it is possible to obtain the likelihood function of an event sequence $A_t$ as:

6 $$\begin{aligned} L(\theta ) = \prod _{m=1}^M\frac{\exp \left\{ \theta ^\top s(i_m,j_m,y_m)\right\} }{\sum _{j \in N_m\setminus \{i\}}\exp \left\{ \theta ^\top s(i_m,j,y_m)\right\} }. \end{aligned}$$

## Dynam specification

The multiple relational mechanisms associated to different aspects of the network dependence structure are captured by network statistics. Their importance stems from the fact that they convey all the information about the relational structure of the data. Stadtfeld and Block (2017) and Stadtfeld et al (2017) provide an overview of the statistics that can be used in both directed and undirected DyNAMs. The main feature of DyNAM statistics is their time-dependence as opposed to those employed in SAOMs (Fritz et al 2020).

Generally speaking, we can classify these statistics into three main classes:

1. statistics defined at the node level - e.g., out-degree;
2. statistics defined at the dyadic (event) level - e.g., inertia;
3. statistics defined at the extra-dyadic statistics - e.g., transitive triads.

In this paper, the covariate information that we will be using in our data analysis is fixed and not changing over time therefore we explicitly model endogenous processes of the data (Stadtfeld and Block 2017). We will analyse directed relational events, hence we will refer to the corresponding network statistics for the model specification. In particular, we define the in-degree and out-degree statistics (belonging to the first class); the inertia statistic (belonging to the second class); and covariate-based nodal statistics (belonging to the first class) and matching statistics (belonging to the second class). However, the reader is referred to Amati et al (2019) for a list of effects.

**Table 1:** Description of the directed network statistics used in our analysis

	Statistic	Sub-graph	Sub-model
in-degree	$\sum _k {\mathbb {I}}[x_{ki}>0]$		Rate/Choice
Out-degree	$\sum _k{\mathbb {I}}[x_{ik}>0]$		Rate/Choice
Inertia	$s_1(i,j,y) = x_{ij}$		Choice
Nodal attribute	$r_3(i,y) = {\mathbb {I}}_{z(i,y)}$		Rate
Matching attribute	$s_2(i,j,y) = {\mathbb {I}}_{z(i,y) = z(j,y)}$		Choice

The in-degree statistic (first row of Table 1) counts the number of incoming ties to node *i* happened in the past; in its formulation *x* is the weighted and time-dependent network and ${\mathbb {I}}[ \cdot ]$ is the indicator function. The out-degree statistic (second row of Table 1) counts the number of outgoing ties from node *i* happened in the past. In the rate sub-model, defined in Equation (3), the in-degree statistic $r_1(i,y)$ measures the tendency of a node *i* to send ties provided that *i* has a high number of incoming ties and the out-degree statistic $r_2(i,y)$ measures the tendency of *i* to receive ties given that *i* has a high number of outgoing ties. In the choice sub-model, defined in Equation (5), the in-degree statistic $s_1(j,y)$ measures the tendency to create a tie $i \rightarrow j$ when *j* has a high number of incoming ties and the out-degree statistic $s_2(j,y)$ measures the tendency to create a tie $i \rightarrow j$ when *j* has a high number of outgoing ties. The inertia statistic, (third row of Table 1) can only be defined for the choice sub-model as it represents a dyadic effect. It measures the tendency to create a tie $i \rightarrow j$ when the same tie happened before. In simple terms, it is the count of events $i \rightarrow j$ happened up to time *t*.

Model statistics may also be defined dependent upon exogenous covariates which can be nodal, dyadic or higher order, i.e., the *z* objects described at the beginning of Sect. 3. In the second last row of Table 1 we define a nodal attribute statistic referring to the sender node in the context of the rate sub-model. It captures the likeliness for a sender of getting active if they show such attribute.

The dyadic matching attribute statistic captures the tendency of both sender and receiver to share the same nodal covariate value (last row of Table 1). In simple terms, it equals one when both *i* and *j* have the same covariate.

## Bayesian parameter estimation

The classical estimation method for DyNAMs is based on maximum likelihood estimation by means of numerical optimization methods such as Fisher scoring or Newton-Raphson procedure (Stadtfeld and Block 2017).

The Bayesian approach to complex statistical models consists of a coherent inferential framework for the analysis of all the uncertainties by constructing a fully-probabilistic interpretation of the model parameters via posterior distributions. Expert information can be included so as to guarantee better accuracy in the estimation by means of informative priors. As a result, Bayesian techniques in social network analysis has proven to bring advantages in terms of estimation accuracy compared to their classical counterparts (see, for example, Koskinen and Snijders (2007); Caimo and Friel (2011)).

Being DyNAMs a two-step process (Equation 2), their Bayesian estimation procedure is carried out independently for each sub-model. In this paper the estimation is based on a Monte Carlo algorithm sampling from the target posterior of the parameters of the rate and choice sub-models $\beta , \theta$ given the data *y*:

$$\begin{aligned} p(\beta , \theta \mid y)= \frac{p(\beta , \theta ) \ p(y \mid \beta , \theta )}{\int \int p(\beta , \theta ) \ p(y \mid \beta , \theta )\;d\beta d\theta }, \end{aligned}$$

where the numerator is the product between the prior $p(\beta , \theta )$ and the DyNAM likelihood $p(y \mid \beta , \theta )$ and the denominator is the intractable marginal likelihood or model evidence representing the probability of the data *p*(*y*). The DyNAM likelihood can be written as the product of the two sub-model likelihoods defined in Equations (4) and (6):

$$\begin{aligned} p(y \mid \beta , \theta ) = L^{\mu }(\beta ) \times L(\theta ). \end{aligned}$$

A Monte Carlo procedure (described in Algorithm 1) is adopted in order to carry out inference for the target parameter posterior distribution. 
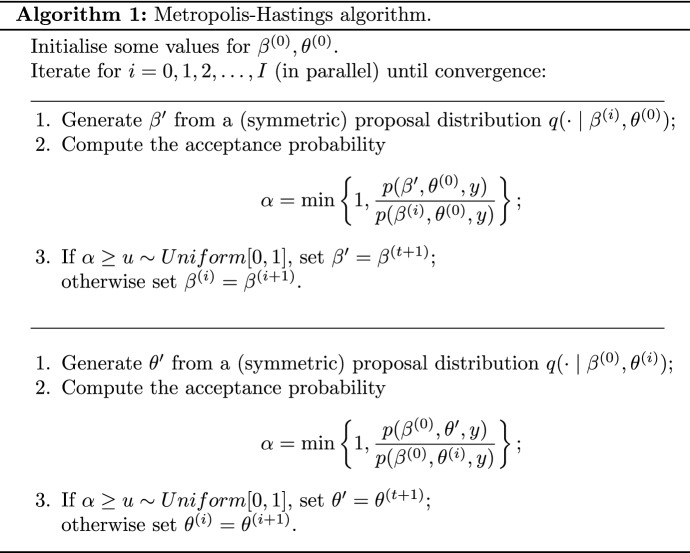


Given the separable nature of the two DyNAM sub-models the two sets of parameters $\beta$ and $\theta$ can be estimated by two parallel MCMC procedures. A convenient choice of the proposal distribution is a symmetric probability distribution such as the normal distribution. This is the distribution that we use for the example and application in Sect. 6 and Sect. 7 respectively.

## Example: social evolution data

The Social Evolution data source is a well-known relational dataset composed of 84 nodes representing students living in a U.S. student house. It can be retrieved from the **goldfish** package (Stadtfeld and Hollway 2020) where a reduced observed time span is displayed as opposed to the original in order to analyse, in line with Stadtfeld et al (2017), a sequence of phone calls between students in a descriptive fashion. In our example, we show how the uncertainty associated to the relational tendencies of the dynamic event process can be analysed using the posterior distribution of the parameters. The resulting interpretation of our Bayesian estimation approach is straightforward and consistent with the results obtained by Stadtfeld et al (2017). For this example, we introduce some of the statistics as described in Sect. 4 for both the rate and choice sub-models. In spite of this, we refer to both the networks available in the **goldfish** package, namely the phone call activity that includes the communications between students over time, and the friendship network accounting for the students’ social structure.

### Model specification

We define a cumulative number of nine effects of which four influence the general tendency of individuals to make phone calls, while the other five concern the activity to direct them towards certain receivers. These effects are conveyed by means of the model parameters.

The effects for the rate sub-model are:

- Intercept, describing how likely actors are to make phone calls. Namely, how the baseline actor, with no outgoing or incoming calls, is incline to pick up the phone and dial a number (conveyed by the $\beta _0$ parameter).
- In-degree, capturing the tendency to assess whether having received phone calls in the past increases or decreases how active is the sender ($\beta _1$).
- Out-degree, which describes the tendency to get active in relation to having made phone calls in the past ($\beta _2$).
- Out-degree friendship, highlights the question of whether having more friends increases or not the tendency to make a phone call ($\beta _3$).

The statistics included in the choice sub-model are:

- In-degree and Out-degree effects conveyed by $\theta _1$ and $\theta _2$ respectively. In the choice sub-model these effects focus on the receiver. Hence they are respectively trying to answer the following questions: what is the tendency to receive a phone call if the receiver got several calls in the past (in-degree, $\theta _1$)? What is the tendency to receive a phone call if the receiver made several calls in the past (out-degree, $\theta _2$)?
- Inertia (intercept in the choice sub-model) captures the tendency of students to make calls mainly to those whom they called in the past ($\theta _3$). Stadtfeld et al (2017) model this behaviour by means of the out-degree. In fact, a negative estimate value implies that individuals tend not to call those who have a lot of outgoing calls but those whom they most likely called before. However, we believe that using the inertia to capture this relational behaviour would result in a more straightforward interpretation.
- Floor membership statistic models the tendency of individuals living on the same floor to have a higher tendency to call each other. It is a matching statistic as defined in the last row of Table 1 ($\theta _4$).
- Inertia friendship statistic highlights the question of whether students tend to call their friends ($\theta _5$).

The estimation is accomplished by sampling from the target posteriors of the two sub-models via the Metropolis-Hastings sampler (Algorithm 1). There are two common choices for the $\beta$ prior distributions of an exponential regression model (like the DyNAM rate sub-model): one is uniform improper prior distribution, the other is the normal distribution (Ibrahim et al 2014). In this example, we specify a multivariate normal prior for the rate sub-model parameters reflecting our a priori assumption of a small baseline hazard of observing a call event at any time point by setting a negative prior mean for the intercept $\beta _{0}$ which is likely to be compensated by some of the other effects included in the model:

$$\begin{aligned} \beta =\left( \beta _{0}, \beta _{1}, \beta _{2}, \beta _{3}\right) \sim {\mathcal {N}}\left( \left[ \begin{array}{c} -10 \\ 0 \\ 0 \\ 0 \end{array}\right] ,\left[ \begin{array}{cccc} 4 &{} 0 &{} 0 &{} 0 \\ 0 &{} 5 &{} 0 &{} 0 \\ 0 &{} 0 &{} 6 &{} 0 \\ 0 &{} 0 &{} 0 &{} 5 \end{array}\right] \right) . \end{aligned}$$

The prior specification of the choice sub-model parameters (Equation 6) consists of a multivariate normal prior reflecting our prior assumption of a positive inertia friendship effect (positive prior mean for $\theta _{5}$):

$$\begin{aligned} \theta =\left( \theta _{1}, \theta _{2}, \theta _{3}, \theta _{4}, \theta _{5}\right) \sim {\mathcal {N}}\left( \left[ \begin{array}{c} 0 \\ 0 \\ 0 \\ 0 \\ 2 \end{array}\right] ,\left[ \begin{array}{ccccc} 4 &{} 0 &{} 0 &{} 0 &{} 0 \\ 0 &{} 4 &{} 0 &{} 0 &{} 0 \\ 0 &{} 0 &{} 4 &{} 0 &{} 0 \\ 0 &{} 0 &{} 0 &{} 4 &{} 0 \\ 0 &{} 0 &{} 0 &{} 0 &{} 5 \end{array}\right] \right) . \end{aligned}$$

### Results

The covariance matrices of the multivariate normal proposal distribution used in Algorithm 1 are defined as follows:

$$\begin{aligned} \left[ \begin{array}{cccc} 0.01 &{} 0 &{} 0 &{} 0 \\ 0 &{} 0.0007 &{} 0 &{} 0 \\ 0 &{} 0 &{} 0.0007 &{} 0 \\ 0 &{} 0 &{} 0 &{} 0.002 \end{array}\right] \end{aligned}$$

for $$q(\beta ' \mid \beta ^{(i)}),$$ and

$$\begin{aligned} \left[ \begin{array}{ccccc} 0.03 &{} 0 &{} 0 &{} 0 &{} 0 \\ 0 &{} 0.05 &{} 0 &{} 0 &{} 0 \\ 0 &{} 0 &{} 0.1 &{} 0 &{} 0 \\ 0 &{} 0 &{} 0 &{} 0.1 &{} 0 \\ 0 &{} 0 &{} 0 &{} 0 &{} 0.7 \end{array}\right] \end{aligned}$$

for $q(\theta ' \mid \theta ^{(i)}).$ The tuning of the parameters is performed so as to achieve an average acceptance rate of 0.23 and 0.25 for the rate and choice sub-models respectively (Gelman et al 1997).

In Table 2, the posterior parameter summaries are displayed. All the results are in line with what Stadtfeld et al (2017) reported. The negative values corresponding to the highest posterior density interval for the intercept $\beta _0$ confirm that, at baseline, a student who has not received or made phone calls in the past nor has friendship ties with other students will hardly make a phone call. All the rate effects ($\beta _1$, $\beta _2$ and $\beta _3$) are positive signalling that the sender gets more active when they made and received calls and if they have friendship connections. Stadtfeld et al (2017) did not include the in-degree statistic in their models, however the positive effect estimate associated to the parameter $\beta _1$ has a straightforward interpretation as any effect increasing the activity of the sender in the past increases their tendency to be active.

**Table 2:** Posterior parameter means and corresponding 95% credible intervals for the Bayesian DyNAM analysis of the Social Evolution Data

Parameter	Mean [95% credible interval]
*DyNAM rate sub-model*	
$\beta _0$ (intercept)	$-15.35$ [$-14.47; -16.20$]
$\beta _1$ in-degree	0.081 [0.071; 0.091]
$\beta _2$ out-degree	0.018 [ 0.017;0.019]
$\beta _3$ out-degree friendship	0.392 [0.357;0.434]
*DyNAM choice sub-model*	
$\theta _1$ in-degree	-0.003 [-0.016; 0.010]
$\theta _2$ out-degree	0.007 [-0.009; 0.021]
$\theta _3$ inertia	0.276 [0.231; 0.324]
$\theta _4$ floor membership	0.099 [-0.220 ; 0.412]
$\theta _5$ inertia friendship	3.08 [2.760 ; 3.412]

The results of the choice sub-model show that the inertia effect ($\theta _3$) is positive, representing the fact that students tend to call those whom they called in the past. In particular, the inertia effect involving students with friendship links ($\theta _5$) is the main effect explaining the behaviour of the choice sub-model. The in-degree effect ($\theta _1$), not included in Stadtfeld et al (2017) and the out-degree effect ($\theta _2$) and the floor membership ($\theta _4$) do not seem to be significant in explaining the receivers’ behaviour. It is important to notice that by introducing the inertia effect ($\theta _3$), we already partially account for the out-degree effect between students that tend to communicate to each other frequently. This translates into a slightly different interpretation of the overall degree based effects, which merely represents the tendency of two students to contact other students (but not necessarily the same set of receiving students) with an higher number of incoming and outgoing calls.

## Application: South Korean COVID-19 patient movement data

The raw dataset has been processed in order to obtain a directed network of movements of infected individuals inside South-Korea, where the nodes represent the municipalities where the individuals are located when they get active and the events consist of their movements between two different locations. The nodes portrait, where possible, a division of South Korea at municipal level or at district/country level given the peculiar municipal division of cities in South Korea. The total number of nodes available in the dataset is 210 of which 27 are either only sending or receiving municipalities. The total number of events occurring from January 22, 2020, to March 25, 2020, is 8, 093 of which 3, 170 are no-loops events, namely movements directed outside the senders’ municipality that cannot end in the same municipality.

It is important to notice that at the beginning of the pandemic the distribution of the infected individuals in South Korea was not proportional to the distribution of the population, i.e., the largest clusters were not necessarily localised in the largest municipalities and vice versa. This is clearly shown in Table 3 where the municipalities within the Seoul region account for about $20\%$ of the overall South Korean population but they represent more than $45\%$ of the overall movement activity included in the dataset. On the other hand, in Gyeonggi-do, the most populated area, municipalities are contributing for about the $6\%$ of the movements. Moreover, the identity of the infected patients moving between municipalities is clearly not available so it is not possible to determine whether a movement from a certain municipality to another was actually made by a patient living in one of the two.

**Table 3:** Percentage of population, and cumulative proportion of out-going and in-coming movements for each South Korea region

Region	Population	Out-going movements	In-coming movements
Seoul	20.4%	46.2%	46.5%
Busan	7.3%	10.9%	11.1%
Incheon	5.2%	10.7%	10.6%
Gyeonggi-do	26.5%	6.5%	6.8%
Daegu	5.1%	5.7%	6.8%
Gyeongsangbuk-do	5.3%	5.4%	3.9%
Chungcheongnam-do	4.1%	4%	4%
Gyeongsangnam-do	6.8%	2.2%	2.1%
Gwangju	2.8%	1.7%	1.7%
Chungcheongbuk-do	3.1%	1.6%	1.7%
Ulsan	0.0%	1.5%	1.1%
Daejeon	2.9%	1.2%	1.4%
Gangwon-do	3.1%	1.0%	0.9%
Jeollanam-do	3.8%	0.6%	0.6%
Jeollabuk-do	3.7%	0.4%	0.4%
Sejong	0.0%	0.4%	0.0%

**Fig. 1:**
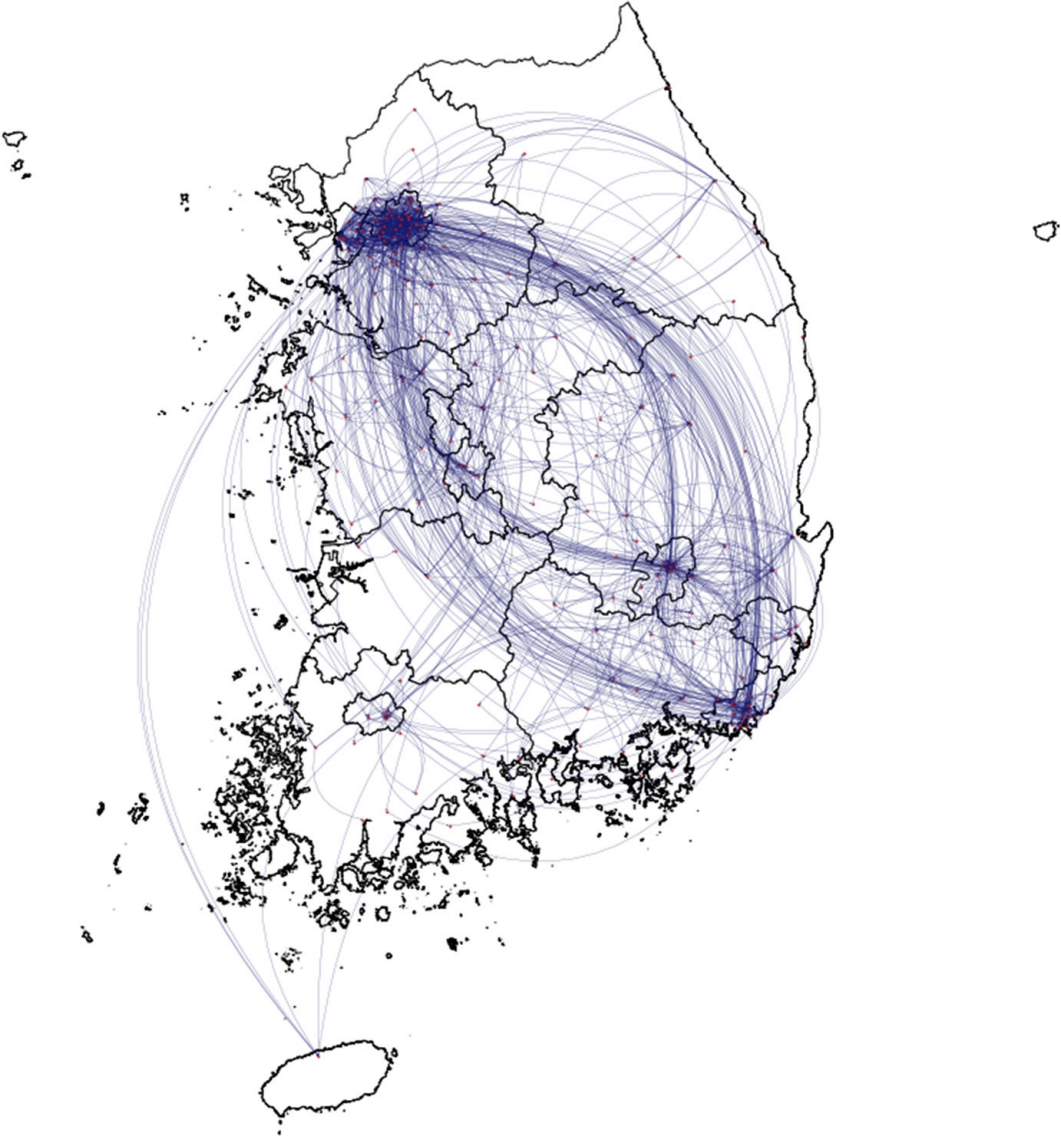
Graphical representation of all patient movements in South Korea, the red nodes represent the municipalities from and to which they move, and the arrows the events between the nodes

**Fig. 2:**
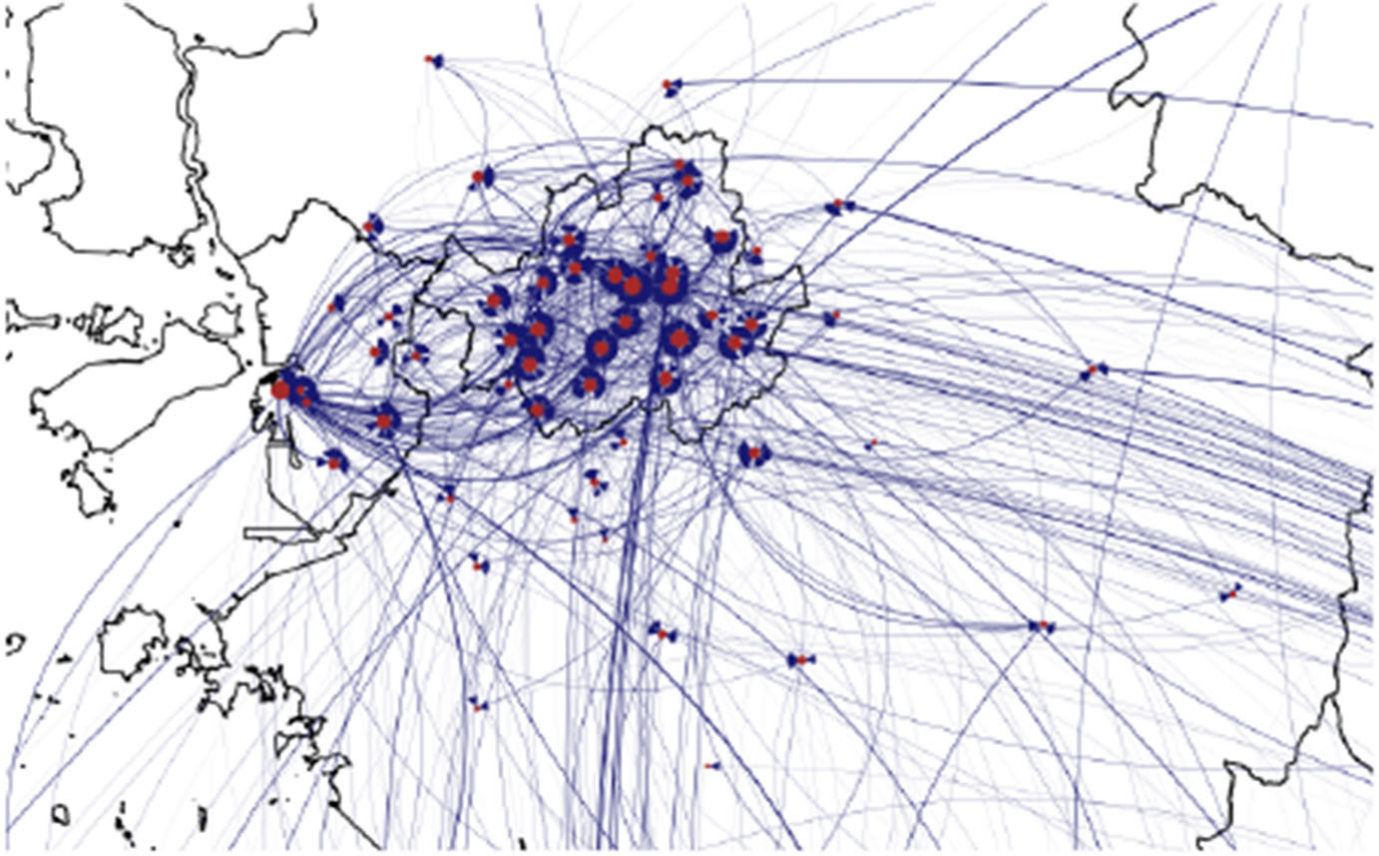
Patient movements within Seoul region and its surroundings

Figure 1 gives a graphical representation of all the events in the two months period. On a first visual inspection of the overall relational event graph, it seems clear that most of the patient movements start from or end to the municipalities of the largest cities, like Seoul, Incheon (see Fig. 2) and Daegu. Therefore, policies focusing on widespread testing and public notice of movements of those infected in these areas were particularly successful in the prevention of the virus diffusion.

### Model specification

In our empirical analysis, we specify in the rate sub-model the general tendency of cities to create a tie and hence of patients to move outside of it, whereas in the choice sub-model we specify the propensity to select a given municipality as the destination of a patient movement. Given the directed nature of the events of the network under study, it is important to capture both tendencies described above to enhance the interpretation of its relational dynamics. This is best achieved by means of DyNAMs and their interdependence assumptions between the sub-models.

The effects, conveyed by the $\beta$ parameters, characterizing the rate function of the sender $\mu _i(y,\beta )$ are:

- Intercept; modelling the baseline propensity of observing the movement of an infected individual (conveyed by $\beta _0$).
- In-degree effect; modelling how the propensity to move is affected by a high number of incoming movements towards the sender municipality in the past ($\beta _1$).
- Out-degree effect; modelling how the propensity to move is affected by a high number of outgoing movements from the sender municipality in the past ($\beta _2$).
- Region effect; a nodal attribute equalling 1 when the sender municipality comes from a certain region and 0 otherwise. It measures whether movements coming from a municipality of a certain region have a tendency of moving inside or outside that region. This effect has been used for Seoul ($\beta _3$), Busan ($\beta _4$), Incheon ($\beta _5$), and Daegu ($\beta _6$).

As for the effects influencing the tendency to choose the destination of the movement, namely the municipalities towards which a patient goes, we control for three main effects (inertia, in-degree and out-degree). All these network statistics are introduced for modelling both the overall network and for that part of the network consisting of the ties within the major regions only. The latter are coded by means of matching statistics counting the number of ties between senders and receivers belonging to the same area. The effects, conveyed by the $\theta$ parameters, are:

- In-degree effect: patients tend to go to cities which had more incoming events in the past ($\theta _1$).
- Out-degree effect: patients tend to go to cities from where a high number of movements originated in the past ($\theta _2$).
- Inertia effect: if patients tend to replicate the same route, hence if they tend to visit a location they visited in the past ($\theta _3$).
- In-degree effect for the Seoul region ($\theta _4$).
- Out-degree effect for the Seoul region ($\theta _5$).
- Inertia effect for the Seoul region ($\theta _6$).
- Inertia effect for the Busan region ($\theta _7$).
- Inertia effect for the Incheon region ($\theta _8$).
- Inertia effect for the Daegu region ($\theta _9$).

As for the previous example, the estimation is accomplished by sampling from the target posteriors of the two sub-models via the Metropolis-Hastings sampler (Algorithm 1). The prior specification of the sub-model parameters (Equation 4, 6) consists of multivariate normal priors:

$$\begin{aligned} \beta =\left( \beta _{0}, \beta _{1}, \beta _{2}, \beta _{3}, \beta _{4}, \beta _{5}, \beta _{6} \right) \sim {\mathcal {N}}\left( \left[ \begin{array}{c} -4 \\ 0 \\ 0 \\ 4 \\ 4 \\ 4 \\ 4 \end{array}\right] ,\left[ \begin{array}{ccccccccc} 2 &{} 0 &{} 0 &{} 0 &{} 0 &{} 0 &{} 0 \\ 0 &{} 4 &{} 0 &{} 0 &{} 0 &{} 0 &{} 0 \\ 0 &{} 0 &{} 4 &{} 0 &{} 0 &{} 0 &{} 0 \\ 0 &{} 0 &{} 0 &{} 2 &{} 0 &{} 0 &{} 0 \\ 0 &{} 0 &{} 0 &{} 0 &{} 2 &{} 0 &{} 0 \\ 0 &{} 0 &{} 0 &{} 0 &{} 0 &{} 2 &{} 0 \\ 0 &{} 0 &{} 0 &{} 0 &{} 0 &{} 0 &{} 2 \end{array}\right] \right) . \end{aligned}$$

The prior specification of the choice sub-model parameters (Equation 6) consists of independent normal priors centered at 0 with a variance of 2:

$$\begin{aligned} \theta =\left( \theta _{1}, \theta _{2}, \theta _{3}, \theta _{4}, \theta _{5}, \theta _{6}, \theta _{7}, \theta _{8}, \theta _{9}\right) \sim {\mathcal {N}}\left( \mathbf{0}, 2\mathbf{I_9}\right) , \end{aligned}$$

where $\mathbf{I_9}$ is the $9 \times 9$ identity matrix.

### Results

The interpretation of the DyNAM posterior results (displayed in Table 4) can be drawn separately for each sub-model as the two sub-models are conditionally independent given the process state. The MCMC algorithm implemented in this application made use of independent normal proposal distributions with standard deviations of 0.1, 0.0018, 0.002, 0.15, 0.5, 0.5, 0.5 for the rate sub-model parameters $\beta _0, \cdots , \beta _6$ and of 0.0009, 0.0009, 0.01, 0.005, 0.007, 0.05, 0.4, 0.3, 0.7, for the choice sub-model parameters $\theta _1, \cdots , \theta _9$ so as to reach an average acceptance rate of about 0.21 for the rate sub-model and about 0.25 for the choice sub-model.

As the descriptive inspection suggests, the overall tendency to move between municipality is strong, hence the time necessary for a patient to leave a municipality to reach another one is $1/ \exp (\beta _0) \approx 16$ minutes. The in-degree effect ($\beta _1$) is negative but with a small magnitude. The sender tendency of a municipality to experience an out-going movement is slightly reduced when the municipality has a high degree of in-coming movements in the past. The out-degree effect ($\beta _2$) is associated to a positive estimate, meaning that senders’ waiting time tends to reduce if they got active in the past.

We also controlled for the membership effect associated to the region of the sender municipality ($\beta _3$,$\beta _4$,$\beta _5$,$\beta _6$). The results show a positive tendency to observe an outgoing movement event if the sender municipality belongs to one of the controlled regions. This result is in line with our preliminary descriptive analysis, as the majority of events generate within such areas. The similar magnitude of these parameters indicates that the size of the sending municipalities does not signal an impact on the tendency to move from it. Even if varying in size, the regions have similar trends in terms of the sender effect.

**Table 4:** Posterior parameter means and corresponding 95% credible intervals for the Bayesian DyNAM analysis of the South Korea COVID-19 patient movement data

Parameter	Mean [95% credible interval]
*DyNAM rate sub-model*	
$\beta _0$ (intercept)	-6.481 [-6.934; -5.798]
$\beta _1$ in-degree	-0.004 [-0.006; -0.002]
$\beta _2$ out-degree	0.008 [ 0.005;0.011]
$\beta _3$ Seoul membership	5.617 [4.927;6.075]
$\beta _4$ Busan membership	5.541 [4.157;6.071]
$\beta _5$ Incheon membership	5.602 [4.901;6.073]
$\beta _6$ Daegu membership	5.532 [3.959;6.071]
*DyNAM choice sub-model*	
$\theta _1$ in-degree	0.099 [0.089; 0.108]
$\theta _2$ out-degree	-0.083 [-0.108;-0.074]
$\theta _3$ inertia	0.658 [0.549;1.523]
$\theta _4$ out-degree Seoul	-0.063 [-0.075;-0.005]
$\theta _5$ in-degree Seoul	0.062 [0.0489;0.084]
$\theta _6$ inertia Seoul	-0.281 [-1.173;-0.177]
$\theta _7$ inertia Busan	-3.14 [-5.642;-1.430]
$\theta _8$ inertia Incheon	0.119 [-0.246;0.233]
$\theta _9$ inertia Daegu	-0.186 [-0.842;-0.020]

For the choice sub-model, we observe opposite tendencies for the in-degree and out-degree effects ($\theta _1$, $\theta _2$) with respect to the ones observed in the rate sub-model, and a strong positive parameter value for the inertia ($\theta _3)$. This means that municipalities that have a high incoming number of ties are more likely to be receivers and those with a high number of outgoing ties are less likely to be receivers, implying that movements between municipalities are directed and unidirectional, and therefore not cyclic. The parameter associated to the inertia effect ($\theta _3$) has an high positive value meaning that routes performed by infected subjects tend to be frequent over time. We also included the same kind of effects as the ones described above but weighted by the matching covariate-based statistic for the Seoul region ($\theta _4$, $\theta _5$, $\theta _6$). We observe that the only parameter behaving in an opposite way with respect to the whole country parameters is the inertia ($\theta _6$) that is negative. We can therefore say that movements within the Seoul region still show non-cyclical patterns, in fact, the infected individuals movements’ propensity was higher towards receiving municipalities of the Seoul area and lower towards sending municipalities. The negative Seoul inertia effect instead points out that movements between municipalities located in that region lack regularity hence presenting a more sparse behavior. To put it in other words, sending and receiving municipalities patterns do not replicate over time. Therefore, we may infer that the spread of the virus within the Seoul region was more random and rather harder to be depicted. Moreover, apart from the Incheon inertia effect ($\theta _8$) whose $95 \%$ credible interval includes 0, the inertia effects within Busan ($\theta _7$) and Daegu ($\theta _9$) are negative. The negative sign of such inertia parameters decreases the tendency to replicate past movements, hence we can conclude that patients movement patterns within the largest South Korean regions are irregular and noisy.

## Conclusions

In this paper we proposed a Bayesian estimation approach for dynamic network actor models in order to analyse the relational dynamics of the COVID-19 patient movements in South Korea at the beginning of 2020.

Our results provided strong evidence to the fact that: the baseline tendency of infected patients to leave a municipality is sparse but frequent; the largest regions have a higher tendency to generate events suggesting that outgoing movements from them should be deterred especially at early stages in order to reduce the spread of the disease; new movement events tend to be created from highly active departing municipalities regardless of their population size; at country level, movement events tend to be directed consistently towards the same municipalities and popular destinations. Patient movements within the Seoul region are significant but more random than in the rest of the country given the absence of clear patterns in the sequence of the movement events. Moreover, region-based effects in highly density populated regions are random suggesting harder prevention schemes.

Although Bayesian inference has been recently proposed for most statistical network models (see, for example, Koskinen and Snijders (2007); Caimo and Friel (2011); DuBois et al (2013)), to our knowledge, this is the first fully probabilistic approach developed for dynamic network actor models.

The Bayesian framework is particularly advantageous in this context as it allows the researcher to include their knowledge about network effects via prior specification and draw conclusions based on posterior estimates of the parameters associated to those effects.

The Bayesian estimation procedure, carried out by a Metropolis-Hastings algorithm, delivered samples from the posterior distribution of the model parameters for both the DyNAM rate and choice sub-models. These yielded interesting insights about the dynamics of the relational process involved in the South Korea COVID-19 patient movement data as they captured important effects of the sender and receiver structure of the network.

Further work still needs to be done in order to develop a comprehensive Bayesian approach for DyNAMs. One of the most important future directions consists in developing efficient model choice procedures for the selection of the main network effects to include in the DyNAM sub-models and making practical goodness of fit tests using posterior predictive distributions.

## Footnotes

1 Fleming S. South Korea’s Foreign Minister explains how the country contained COVID-19. World Economic Forum COVID Action Platform. March 31, 2020. https://www.weforum.org/agenda/2020/03/south-korea-COVID-19-containment-testing/. Accessed June 11, 2020.

2 Fisher M, Sang-Hun C. How South Korea Flattened the Curve. New York Times. Published March 23, 2020. Updated April 10, 2020. https://www.nytimes.com/2020/03/23/world/asia/coronavirus-south-korea-flatten-curve.html. Accessed June 11, 2020.

3 COVID-19 National Emergency Response Center, Epidemiology & Case Management Team, Korea Centers for Disease Control & Prevention. Contact transmission of COVID-19 in South Korea: Novel investigation techniques for tracing contacts. Osong Public Health and Research Perspectives. 2020;11(1):60-63. http://doi.org/10.24171/j.phrp.2020.11.1.09. Accessed June 11, 2020.
